# Predictive Value of Lung Ultrasound Combined With ACEF Score for the Prognosis of Acute Myocardial Infarction

**DOI:** 10.1002/clc.70077

**Published:** 2025-02-03

**Authors:** Ziheng Lun, Jiexin He, Ming Fu, Shixin Yi, Haojian Dong, Ying Zhang

**Affiliations:** ^1^ Department of Cardiovascular Medicine Guangdong Cardiovascular Institute, Guangdong Provincial People's Hospital, Guangdong Academy of Medical Sciences Guangzhou Guangdong Province China; ^2^ Department of Cardiovascular Medicine Guangdong Provincial People's Hospital, Guangdong Academy of Medical Sciences Guangzhou Guangdong Province China

**Keywords:** ACEF score, acute myocardial infarction, B‐line, lung ultrasound

## Abstract

**Background:**

Lung ultrasound (LUS) and the ACEF score (age, creatinine, and ejection fraction) have been shown to be pivotal in predicting an unfavorable prognosis in acute myocardial infarction (AMI).

**Hypothesis:**

The aim of this study is to investigate the prognostic value of LUS combined with ACEF score in AMI.

**Methods:**

The ACEF score and the total number of B‐lines in eight thoracic regions of LUS were calculated. Adverse events were recorded during hospitalization and follow‐up, defined as all‐cause death and other cardiovascular events. Multivariate logistic regression identified predictors of adverse events during hospitalization. Multivariate Cox regression identified predictors of adverse events during follow‐up.

**Results:**

We enrolled 204 patients. The B‐lines (adjusted OR 1.08, [95% CI: 1.03–1.13], *p* < 0.01) and the ACEF score (adjusted OR 2.71 [95% CI: 1.07–6.81], *p* < 0.05) independently predicted adverse events during hospitalization. The C‐index values were 0.81 (*p* < 0.01) for the ACEF score, 0.81 (*p* < 0.01) for LUS, and 0.86 (*p* < 0.01) for their combination. One hundred seventy‐one patients were followed up for 12 months (IQR, 8.13–15.93). Both the B‐lines (adjusted HR 1.06 [95% CI: 1.03–1.09], *p* < 0.05) and the ACEF score (adjusted HR 1.95 [95% CI: 1.10–3.43], *p* < 0.05) remained associated with an increased risk of adverse events during follow‐up. The C‐index values were 0.74 (*p* < 0.01) for the ACEF score, 0.73 (*p* < 0.01) for LUS, and 0.80 (*p* < 0.01) for their combined predictive ability.

**Conclusions:**

The B‐lines and ACEF score are associated with adverse events in AMI patients. When combined, they provide increasing value in assessing the risk of adverse events, which has significant implications for risk stratification.

## 1 Introduction

Reperfusion therapy has demonstrated substantial efficacy in reducing mortality among acute myocardial infarction (AMI) patients. Nevertheless, some patients still experience unfavorable outcomes. Notably, AMI complicated by heart failure (HF) remains the leading contributor to increased mortality rates. Consequently, early identification of high‐risk patients and refined risk stratification for AMI are pivotal in informing treatment decisions and optimizing clinical results [1, 2].

The Killip classification [3] has been primarily used to assess the severity of pulmonary edema through pulmonary auscultation in AMI patients [4, 5]. Notwithstanding, pulmonary auscultation exhibits poor sensitivity and specificity, and it cannot promptly identify pulmonary edema. Consequently, this limitation reduces the precision of the Killip classification [6].

Lung ultrasound (LUS), a noninvasive diagnostic tool, demonstrates heightened sensitivity in diagnosing and evaluating pulmonary edema by detecting and quantifying B‐lines. Ultrasound generates intense reverberation at the junction of gas and water. The comet tail sign, emitted perpendicularly from the pleural line, is termed as B‐line. The occurrence of over three B‐lines across two or more intercostal spaces signifies a diagnostic condition known as interstitial syndrome [7]. The emergence of B‐lines is associated with the thickening of alveolar septa and pulmonary edema, while the count of B‐lines correlates with the signs of extravascular lung water observed on chest X‐ray and NT‐proBNP levels [8, 9]. In patients with cardiogenic pulmonary edema, the number of B‐lines is closely related to the severity of pulmonary edema [10, 11, 12]. Daniel A Lichtenstein et al. studied the use of LUS in the diagnosis of acute respiratory failure and proposed the BLUE protocol, which has a sensitivity of 97% and a specificity of 95% in the diagnosis of pulmonary edema [11]. Shunichiro Nakao et al.'s study further revealed that LUS exhibits sensitivity of 92.5% and specificity of 85.7% in diagnosing HF, outperforming chest radiography, which showed sensitivity and specificity of 63.6% and 92.9% respectively. Notably, LUS demonstrated superior sensitivity compared to chest radiography, particularly in the elderly population [13]. A study exploring the relationship between the number of B‐lines upon hospital admission and in‐hospital mortality in patients with acute heart failure (AHF) found that individuals with ≥ 19 B‐lines faced a fourfold heightened risk of in‐hospital mortality (HR 4.38; 95% CI: 1.37–13.95, *p* < 0.01). This suggests a direct association between the number of B‐lines in AHF patients and their in‐hospital mortality risk [14]. Similarly, a prospective investigation by Elke Platz et al. indicated that an increased number of B‐lines at hospital admission heightened the risk of adverse events during hospitalization for AHF patients. Moreover, a higher count of B‐lines at discharge was predictive of a greater risk of rehospitalization or all‐cause death due to heart failure. Notably, the number of B‐lines at discharge emerged as a superior predictor of short‐term adverse events [15]. Recent studies indicate that an increasing number B‐lines in AMI patients is associated with HF and death during hospitalization [16, 17]. However, the predictive efficacy for the post‐discharge prognosis of patients with AMI remains uncertain due to a paucity of relevant research on this matter.

The Age, Creatinine, and Ejection Fraction (ACEF) score, based on clinically relevant parameters, is endorsed by European guidelines for risk stratification during percutaneous coronary intervention (PCI) in AMI patients [18]. Recent studies highlight the important role of the ACEF score in assessing the prognostic value of AMI patients during follow‐up [19, 20]. It is of significant clinical interest to explore whether the combination of LUS with the ACEF score provides extra worth in evaluating the prognosis of AMI patients. Therefore, the objective of our research is to investigate the worth of LUS in assessing the short‐ and medium‐term prognosis of AMI patients and determine whether LUS, when coupled with the ACEF score, has extra predictive worth in evaluating their prognosis.

## 2 Methods

### 2.1 Study Design and Population

Patients diagnosed with AMI who experienced emergency PCI at Guangdong Provincial People's Hospital between February 2021 and June 2022 were enrolled in this prospective cohort. Inclusion was based on the following: (1) age > 18 years old; (2) diagnosis of suspected AMI, fulfilling the diagnostic requirements of AMI in present guidelines (with or without ST‐segment elevation) [21]. Participants who did not satisfy the inclusion criteria were not included in the research.

The study employed the following exclusion criteria: (1) Pulmonary fibrosis or other severe diseases (such as lung cancer, severe emphysema, or severe pleural effusion) that hindered lung ultrasound image acquisition; (2) Patients who did not undergo lung ultrasound within 48 h on admission; (3) Acute myocardial infarction with confirmed nonobstructive coronary artery; (4) Age greater than 80 years old. Elderly patients were excluded from the study because they often had multiple chronic diseases, decreased organ function, and poor prognosis. In our study, we initially selected 226 patients who met the inclusion criteria. Based on the exclusion criteria, we eventually included 204 patients. All patients received best drug treatment in accordance with current guidelines [22, 23], and timely and appropriate PCI was executed. Hospital electronic medical records were looked back meticulously, and we obtained follow‐up data through post‐discharge outpatient visits and telephone interviews. At the same time, we recorded the number of vessels with coronary artery stenosis greater than 50% during PCI. All patients experienced venous blood sampling and laboratory tests upon admission, and echocardiography and lung ultrasound (LUS) were performed within 48 h after admission. Our research was agreed with the Ethics Committee of Guangdong Provincial People's Hospital (Ethics No. KY‐Q‐2022‐103‐02). All patients afforded written informed consent.

### 2.2 Echocardiography and Lung Ultrasound

Within 48 h of admission, Patients underwent echocardiography and LUS. Bedside LUS and transthoracic echocardiography were executed by using a Philips 7 C ultrasound device equipped with a 2.5 MHz phased‐array transducer. Left ventricular end‐diastolic and end‐systolic volumes were observed through the apical four‐ and biventricular compartments in accordance with the suggestions of the American Society of Echocardiography (modified Simpson's rule). In the same time, we calculated the left ventricular ejection fraction (EF). On the parasternal long axis view, we measured the anteroposterior diameter of the left atrium (LA), taking a straight line from the anterior wall of the distal aorta to the anterior wall of the LA. We measured the tricuspid annular plane systolic excursion (TAPSE) as recommended. Diastolic function was evaluated using pulsed and tissue Doppler imaging to derive the E/e' ratio from mitral inflow patterns [24]. The LUS was executed simultaneously with the echocardiogram. All patients were positioned on their back. The LUS was conducted using an 8‐zone method, and the number of B‐lines seen in every zone was enumerated [25]. We then aggregated all the number of B‐lines across the 8 zones and divided the patients into two groups based on the severity of lung water detected by LUS [26]: those with B‐lines ≥ 13 were classified as the high B‐line group, while those with B‐lines < 13 were categorized as the low B‐line group. These examinations were conducted by an experienced operator.

### 2.3 Biochemical Analysis and the ACEF Score

We took all peripheral venous blood samples upon admission and collected in sterile tubes holding Ethylene Diamine Tetraacetic Acid (EDTA). The Abbott Architect assay (Abbott Diagnostics, Abbott Park, IL, USA) was using to perform N‐terminal pro‐brain natriuretic peptide (NT‐proBNP) analysis. Additionally, we used the electrochemiluminescence method (Roche Diagnostics) to measure troponin T (TnT) levels. Serum creatinine (Cr) was quantified using the enzyme method (Enzymatic). Furthermore, we apply the following formula to calculate the Age, Creatinine, and Ejection Fraction (ACEF) score: Age/left ventricular ejection fraction + 1 (if creatinine > 176 μmol/L) [18].

### 2.4 Outcome

The main focus of our attention was adverse events occurring during both follow‐up and hospitalization. The definition of adverse events encompassed all‐cause death and other cardiovascular events, including cardiogenic shock, recurrent myocardial infarction, acute HF, repeat revascularization due to myocardial ischemia, and emergency department visits due to worsening chest pain.

The definition of cardiogenic shock was systolic blood pressure < 90 mmHg or the use of vasopressors with signs of peripheral malperfusion. Recurrent myocardial infarction adhered to the updated definition of AMI [1]. The definition of acute onset of HF was dyspnea, increased moist rales in the lungs, the need for intravenous diuretics, or an increase in diuretics. Revascularization due to myocardial ischemia is unplanned and specifically targets the culprit vessels responsible for this myocardial ischemia.

### 2.5 Statistical Analysis

We expressed categorical variables as percentages and counts, while continuous variables were presented as median (Interquartile range [IQR], 25th−75th percentiles) or mean (± SD), appropriately. We examined the B‐line as a continuous variable, specifically summing the B‐line numbers. We utilized a non‐parametric Spearman correlation coefficient analysis to assess the correlation between the NT‐proBNP, ACEF score and total B‐line numbers. The chi‐square test was used to determine if there was a trend in the change in proportions across groups for binary variables. We employed the Chi‐square test to compare the difference of categorical variables between the different B‐line groups. For continuous variables with a normal distribution, we used the ANOVA test, while the rank sum test was applied to continuous variables without a normal distribution.

The primary and secondary endpoints were considered as outcome variables. Logistic regression models (both unadjusted and adjusted) were utilized to analyze the continuous association between the number of B‐lines and ACEF score with the occurrence of adverse events during hospitalization. Cox proportional hazards models (both unadjusted and adjusted) were employed to assess the association between the number of B‐lines and ACEF score with composite events during the 1‐year follow‐up period after patient discharge. Relevant variables were selected from previous studies and included in univariate analysis. Subsequently, variables with statistically significant differences and clinical importance were incorporated into multivariable regression analysis. To prevent overfitting, a limited number of variables were utilized in the analysis. The following covariates were included in the multivariable model: log‐transformed NT‐proBNP concentration at admission, Killip class, diabetes, use of furosemide, and the number of stenosed vessels. The ACEF score and the number of B‐lines were analyzed using receiver operating characteristic (ROC) curves. The area under the curve (AUC) was calculated to assess the predictive value of each variable for adverse events during hospitalization and follow‐up. Kaplan‐Meier survival analysis was performed to assess event‐free survival, and the differences in survival curves were analyzed using the log‐rank test. We reclassified the combination of ACEF score and the number of B‐lines. These groups were based on the combined prediction probability of the two variables. Patients were divided into three risk groups: G1 (low‐risk) with a range of 0%–33.33%, G2 (medium‐risk) with 33.34%–66.66%, and G3 (high‐risk) with 66.67%–100%.

We employed two‐sided significance levels of 0.05 for all analyses and analyzed Data utilizing SPSS (version 25.0.0; IBM Company).

## 3 Result

### 3.1 Baseline Clinical Characteristics

The major clinical data of a total of 204 patients included is shown in Table 1. All included patients underwent lung ultrasonography. Compared with patients having B‐line < 13, we found that patients with B‐line ≥ 13 were older, had lower admission blood pressure, exhibited higher NT‐proBNP levels and ACEF score, and experienced a longer length of hospital stay. Additionally, patients with B‐line ≥ 13 demonstrated lower EF, larger LA diameter and E/e ‘ratio, and common symptoms and signs of HF, including dyspnea, ankle edema, and rales in the lungs. Meanwhile, patients with B‐line ≥ 13 had a higher proportion of Killip II‐IV grades and a tendency to be more likely to have cardiogenic shock and greater use of furosemide (Table 1).

**TABLE 1:** Baseline characteristics of study subjects according to B‐line.

Characteristic	Overall (*n* = 204)	B‐line < 13 (*n* = 140)	B‐line ≥ 13 (*n* = 64)	*p* value
Age (years)	60.5 (54–68)	59 (53–66)	64 (56–71.8)	0.09
male	174 (85.3%)	118 (84.3%)	56 (87.5%)	0.55
STEMI	140 (68.6%)	96 (68.5%)	44 (68.8%)	0.98
length of hospital stay(days)	7 (6–9)	6 (5–8)	9 (7–13)	0.01
Medical history				
Hypertension	100 (49%)	69 (49.3%)	31 (48.4%)	0.91
Diabetes	53 (26%)	32 (22.9%)	21 (32.8%)	0.13
Hyperlipidemia	52 (25.5%)	41 (29.3%)	11 (17.2%)	0.06
Atrial fibrillation	6 (2.9%)	4 (2.9%)	2 (3.1%)	1.00
Smoking (previous or current)	108 (52.9%)	80 (57.1%)	28 (43.8%)	0.07
Admission characteristics				
Systolic blood pressure (mmHg)	121 ± 22.04	126.04 ± 21.26	100.59 ± 20.13	0.01
Diastolic blood pressure (mmHg)	74.43 ± 15	76.96 ± 15.82	69.11 ± 12.02	0.01
dyspnea	45 (22.1%)	20 (14.3%)	25 (39.1%)	0.01
Ankle edema	17 (8.3%)	4 (2.9%)	13 (20.3%)	0.01
Rales in the lungs	56 (27.5%)	24 (17.1%)	32 (50%)	0.01
Previous PCI	16 (7.8%)	10 (7.1%)	6 (9.4%)	0.58
Cardiac shock	15 (7.4%)	3 (2.1%)	12 (18.8%)	0.01
Furosemide	60 (29.4%)	27 (19.3%)	33 (51.6%)	0.01
Spironolactone	55 (27%)	23 (16.4%)	32 (50%)	0.01
Killip classification				0.01
I	133 (65.2%)	106 (75.7%)	27 (42.2%)	
II	36 (17.6%)	23 (16.4%)	13 (20.3%)	
III	16 (7.8%)	7 (5%)	9 (14.1%)	
IV	19 (9.3%)	4 (2.9%)	15 (23.4%)	
Number of vessels				0.21
Single vessel	50 (26.3%)	38 (27.1%)	12 (18.8%)	
Multiple vessels	140 (73.7%)	93 (66.4%)	47 (73.4%)	
Laboratory results				
NT‐proBNP (pg/ml)	1301 (622–3221)	964 (456–1946)	2855 (1381–5319)	0.04
TnT (ng/ml)	2133 (675–5042)	1541 (506–4272)	3328 (777–5618)	0.06
Creatinine (mg/dl)	82 (67–105)	78 (66–98)	95 (69–132)	0.05
Albumin (g/dl)	38 (34–40)	38 (36–41)	35 (32–38)	0.01
Echocardiography				
EF (%)	47 (38–56)	50 (44–60)	38 (31–46)	0.01
LA diameter (mm)	34 (31–39)	34 (31–38)	35 (31–40)	0.02
E/e' ratio	13 (10–16)	12 (10–15)	15 (11–20)	0.01
TAPSE (mm)	20 (18–23)	18 (21–24)	19 (18–21)	0.82
ACEF score	1.27 (1.02–1.8)	1.14 (0.95–1.47)	1.8 (1.32–2.22)	0.01

Abbreviations: ACEF score: Age, creatinine, and ejection fraction score; EF, ejection fraction; LA, left atrium; NT‐proBNP, N‐terminal pro‐brain natriuretic peptide; PCI, percutaneous coronary Intervention; STEMI, ST‐segment elevation myocardial infarction; TnT, troponin T; TAPSE, Tricuspid Annular Plane Systolic Excursion.

### 3.2 Relationship Between B‐Lines, ACEF Score and Cardiac Function Indexes

**FIGURE 1:**
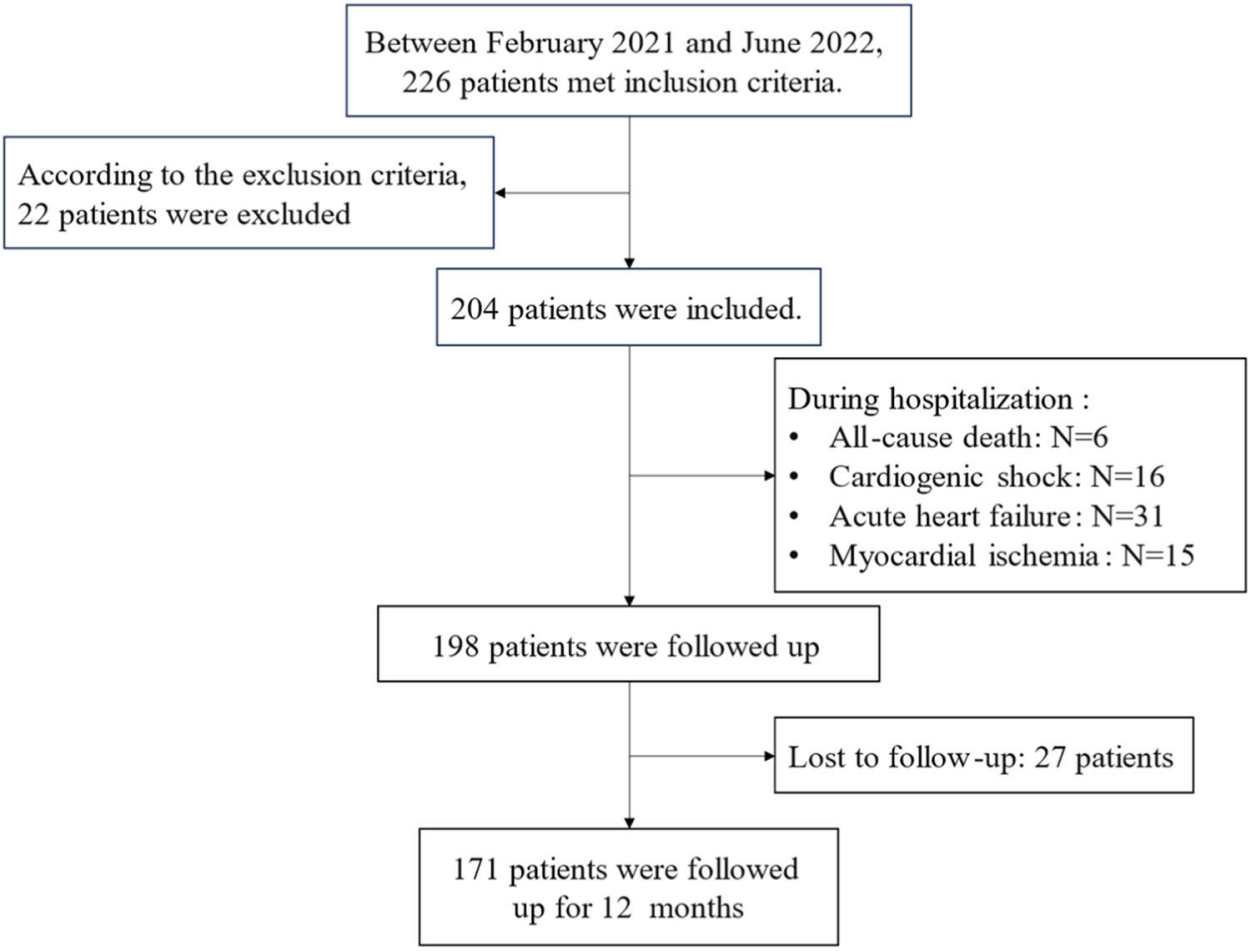
Flow sheet.

The ACEF score positively correlated with NT‐proBNP (r = 0.633, *p* < 0.01), the number of B‐lines (r = 0.404, *p* < 0.01), and E/e' (r = 0.341, *p* < 0.01). Additionally, the number of B‐lines positively correlated with E/e' (r = 0.291, *p* < 0.01) and NT‐proBNP (r = 0.365, *p* < 0.01), but it negatively correlated with ejection fraction (EF) (r = ‐0.431, *p* < 0.01) (Supporting Information S1: Figure 1).

### 3.3 In‐Hospital Adverse Events

The median length of hospital stay was 7 (IQR, 6–9) days. During hospitalization, six patients (2.9%) died from all‐cause, 16 patients (7.8%) experienced cardiogenic shock, 31 patients (15.1%) developed worsening HF, 15 patients (7.3%) had myocardial ischemia, and one patient (0.5%) suffered from acute cerebral infarction. We conducted univariate Logistic regression analysis on various factors to identify covariates suitable for inclusion in the multivariate Logistic regression model (Supporting Information S1: Table 1). In univariate models, ACEF score (OR 9.76 [95% CI: 4.76–20.00], *p* < 0.01) and number of B‐lines (OR 1.13 [95% CI: 1.09–1.18], *p* < 0.01) were statistically significant.

In our multivariate Logistic regression analysis, we discovered that the ACEF score (OR 2.71 [95% CI: 1.07–6.81], *p* < 0.05) and the number of B‐lines (OR 1.08 [95% CI: 1.03–1.13], *p* < 0.01) independently predicted adverse events in patients with AMI during hospitalization (Table 2).

**TABLE 2:** Multivariate analysis during hospitalization and follow‐up.

	Multivariate analysis during hospitalization		Multivariate analysis was performed during follow‐up	
	OR (95% CI)	*p* value	HR (95% CI)	*p* value
Number of B lines	1.08 (1.03–1.13)	0.01	1.06 (1.03–1.09)	0.01
ACEF score	2.71 (1.07–6.81)	0.04	1.95 (1.10–3.43)	0.02
LogNT‐BNP	2.05 (0.82–5.11)	0.12	0.97 (0.52–1.83)	0.93
Killip classification	1.24 (0.80–1.93)	0.34	1.07 (0.79–1.45)	0.68
Diabetes	0.99 (0.38–2.60)	0.99	0.62 (0.30–1.26)	0.19
Furosemide	4.79 (2.02–11.34)	0.01	1.12 (0.56–2.26)	0.75
Number of vessels	0.99 (0.65–1.51)	0.97	1.35 (0.97–1.88)	0.07

Abbreviations: ACEF score: Age, creatinine, and ejection fraction score; HR, hazard ratio; Log NT‐proBNP, log‐transformed N‐terminal pro‐brain natriuretic peptide; OR, odds ratio.

### 3.4 Follow‐Up Adverse Events

A grand total of 171 patients were followed up for a median duration of 12 (IQR, 8.13–15.93) months (Figure 1). During the follow‐up period, we discovered a total of 50 patients (29.2%) experienced adverse events, including six patients (3.5%) with all‐cause death, one patient (0.6%) with recurrent myocardial infarction, 18 patients (10.5%) with myocardial ischemia‐induced revascularization, and nine patients (5.3%) with acute HF. And 17 patients (9.9%) visited the hospital urgently due to aggravation of chest pain. We constructed univariate and multivariate Cox proportional hazards models to confirm factors among the included covariates that could forecast the short‐term and medium‐term prognosis of AMI (Table 2 and Supporting Information S1: Table 1).

In our multivariate Cox proportional hazards model, only the ACEF score (HR 1.95 [95% CI 1.10–3.43], *p* < 0.05) and the total number of B‐lines (HR 1.06 [95% CI: 1.03–1.09], *p* < 0.05) were identified as independent predictors of adverse events during follow‐up (Table 2).

**FIGURE 2:**
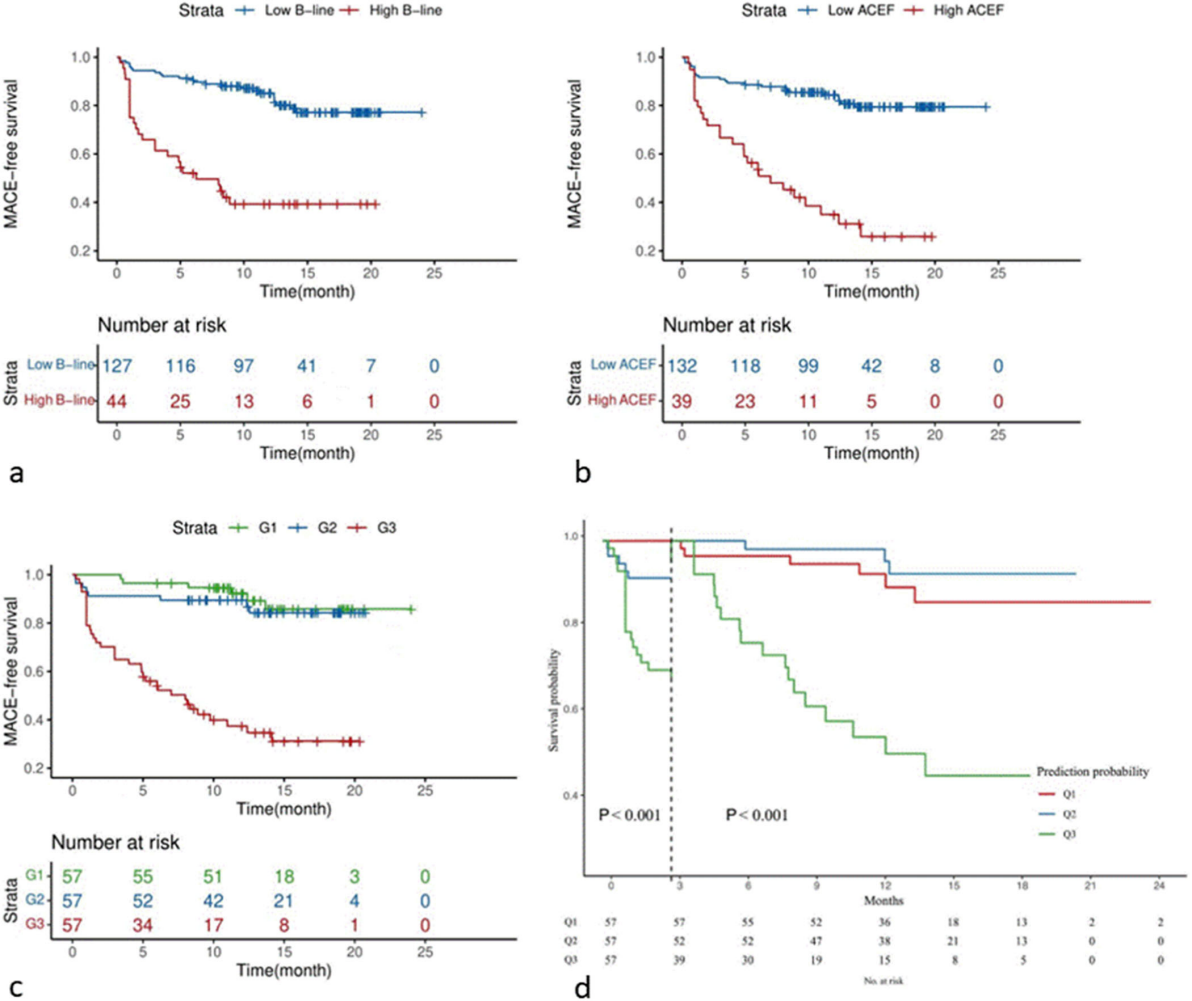
(a) The cumulative incidence of adverse events was categorized based on the number of B‐lines: low B‐line: B‐lines < 13, and high B‐line: B‐lines ≥ 13; (b) The cumulative Incidence of adverse events was grouped according to ACEF score: low ACEF score: ACEF score < 1.69, high ACEF score: ACEF score ≥ 1.69; (c) The cumulative incidence of adverse events was calculated based on tertiles of predictive probability, combining B‐line and ACEF scores. Patients were classified into three risk groups: G1 (low‐risk) with a range of 0%–33.33%, G2 (medium‐risk) with 33.34%–66.66%, and G3 (high‐risk) with 66.67%–100%; (d) Landmark analysis of events before and after 3‐month follow‐up. Patients were classified into three risk groups: Q1 (low‐risk) with a range of 0%–33.33%, Q2 (medium‐risk) with 33.34%–66.66%, and Q3 (high‐risk) with 66.67%–100%.

We performed grouping and Kaplan‐Meier survival analysis based on ACEF score and the number of B‐lines, respectively. Our Kaplan‐Meier curve discovered that the cumulative incidence of adverse events was higher in patients who had more B‐lines (high B‐line vs. low B‐line, 60.7% vs. 22.8%, log‐rank χ^2^ 36.16, *p* < 0.01) (Figure 2a). Correspondingly, patients with a higher ACEF score also had a greater incidence of adverse events (high ACEF score vs. low ACEF score, 74.1% vs. 20.6%, log‐rank χ^2^ 41.86, *p* < 0.01) (Figure 2b). These findings showed that a lower number of B‐lines and a lower ACEF score may identify a subgroup at extremely low risk of adverse outcomes.

We reclassified the combination of ACEF score and the number of B‐lines. Our Kaplan‐Meier curve demonstrated that the cumulative incidence of adverse events was higher in the high‐risk group (high‐risk group vs. medium‐risk group vs. low‐risk group, 68.8% vs. 15.7% vs. 14.2%, log‐rank χ^2^ 58.66, *p* < 0.01) (Figure 2c). Patients in the high‐risk group had a markedly higher risk of adverse events during follow‐up than those in the low‐risk (adjusted HR 5.03 [95% CI: 1.51–16.80, *p* < 0.01]) and the medium‐risk group (adjusted HR 5.72 [95% CI: 2.23–14.63, *p* < 0.01]).

We assessed events within and after 3 months in the landmark analyses (Figure 2d). Within this 3‐month period, we observed no adverse events in the low‐risk group. The cumulative incidence of adverse events among the high‐risk group, medium‐risk group, and low‐risk group was statistically significant (low‐risk group vs. medium‐risk group log‐rank χ^2^ 5.18, *p* = 0.02, low‐risk group vs. high‐risk group log‐rank χ^2^ 21.30, *p* < 0.01, medium‐risk group vs. high risk group log‐rank χ^2^ 8.51, *p* < 0.01). Furthermore, the hazard of adverse events in the high‐risk group was markedly greater than that in the medium‐risk group during the 3‐month follow‐up (adjusted HR 3.62 [95% CI: 1.02–12.92, *p* < 0.05]) (Figure 2d).

### 3.5 Comparison of LUS and ACEF Score in Predicting Adverse Events

**FIGURE 3:**
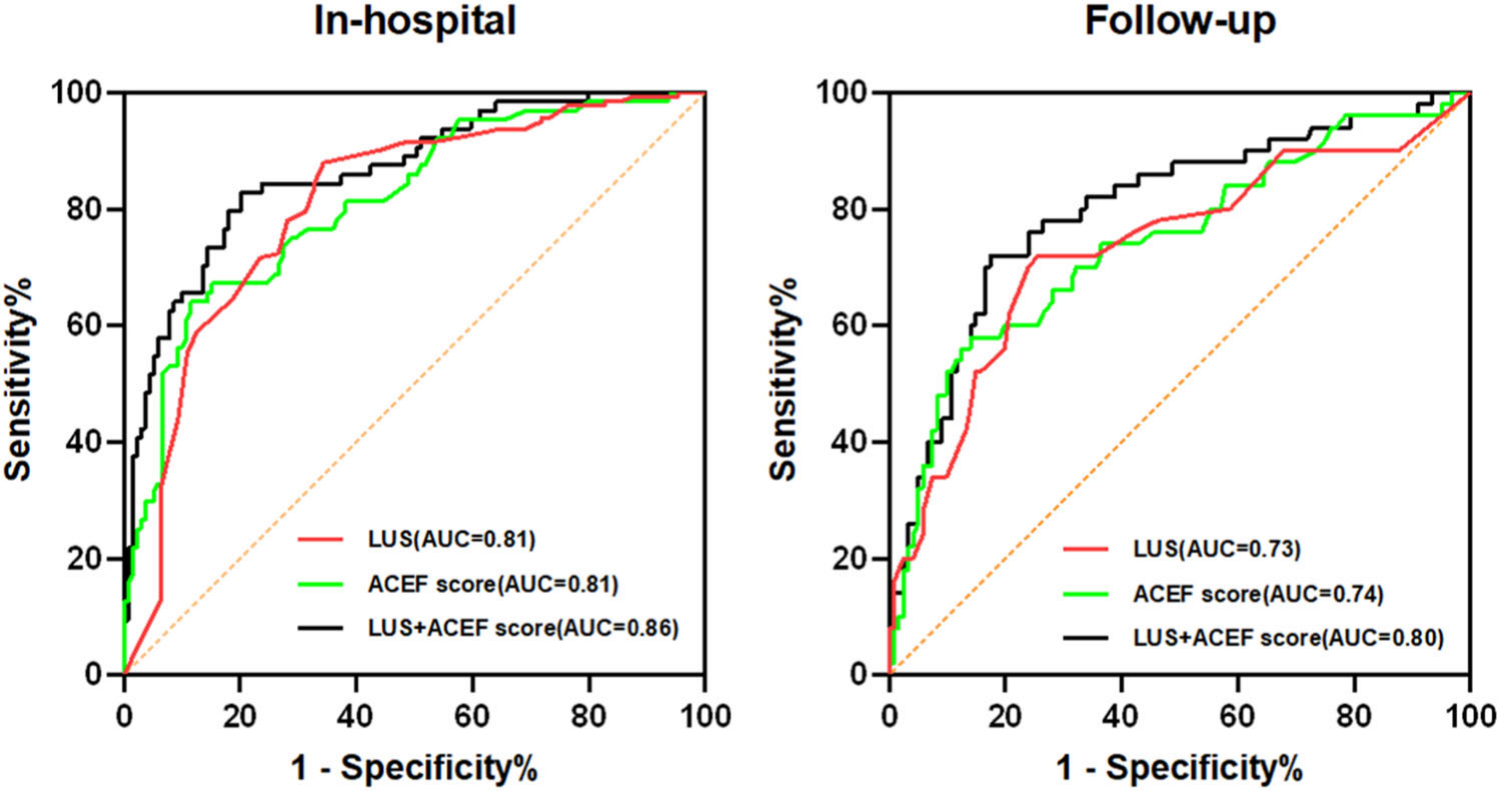
Receiver operating characteristic curves for “LUS”, “ACEF score,” and “LUS + ACEF score” classification to predict the in‐hospital and follow‐up composite outcome. ACEF score, ACEF score classification; AUC, the area under the curve; follow‐up, during 12 months follow‐up; In‐hospital, during the hospitalization; LUS, LUS classification; LUS + ACEF score, the combination of LUS and ACEF score classification.

We employed the receiver operating characteristic (ROC) curve to assess the diagnostic ability of ACEF score and LUS in predicting the clinical outcome of AMI patients during both hospitalization and follow‐up. For predicting in‐hospital adverse events, the area under the ROC curves (AUCs) were 0.81 (*p* < 0.01) for ACEF score, 0.81 (*p* < 0.01) for LUS and 0.86 (*p* < 0.01) for their combination (Figure 3). The ACEF score ≥ 1.69 was the best cut‐off value, with a specificity of 88.5% and a sensitivity of 64.1%. The B‐lines ≥ 13 was the best cut‐off value, with a specificity of 87.9% and a sensitivity of 65.6%.

For predicting adverse events during follow‐up, the AUCs were 0.74 (*p* < 0.01) for ACEF score, 0.73 (*p* < 0.01) for LUS and 0.80 (*p* < 0.01) for their combination (Figure 3). These results indicated that LUS combined with ACEF score was superior to LUS and ACEF score alone in predicting the incidence of adverse events in patients with AMI in the short and medium term. Furthermore, combining the ACEF score with the number of B‐lines has been shown to incrementally enhance the predictive value for adverse events (*p* < 0.05).

## 4 Discussion

This study yielded two main findings. First, B‐lines > 13 were associated with adverse cardiovascular events both in‐hospital and during follow‐up. Second, LUS and the ACEF score demonstrated good predictive value for the short‐term and medium‐term prognosis of patients with AMI. Furthermore, when integrating the ACEF score with the count of B‐lines, it enhanced the prognostic value for evaluation. Therefore, LUS combined with ACEF score can effectively serve as an assessment tool for patients with AMI.

Several studies have demonstrated that B‐lines, as assessed by LUS, hold significant worth in evaluating the short‐term prognosis of patients with AMI. The count of B‐lines in AMI patients has been connected with both mortality and HF during hospitalization [16, 17, 27]. However, it remains unclear whether LUS has predictive value for the medium‐term prognosis of AMI patients, and relevant studies are lacking. Our study demonstrated that the quantity of B‐lines was indeed correlated with the medium‐term prognosis of AMI patients.

AMI patients frequently present with concurrent pulmonary edema [28, 29], which is associated with an unfavorable prognosis. Philippe Gabriel Steg et al. demonstrated that HF is linked to decreased rates of hospitalization and 6‐month survival across all acute coronary syndrome (ACS) subtypes [30]. Our study revealed that a majority of AMI patients exhibited pulmonary edema. However, during hospitalization, most patients were classified as Killip I, highlighting the limited sensitivity of pulmonary auscultation in early identification of pulmonary edema. Notably, patients who have a higher number of B‐lines on imaging not only had elevated NT‐proBNP levels and ACEF scores, but also exhibited reduced ejection fraction (EF). These patients were more likely to experience severe HF symptoms, including dyspnea, pulmonary rales, and low blood pressure. The presence of an elevated quantity of B‐lines in patients with AMI often correlates with a worse prognosis.

Our study revealed that AMI patients treated with furosemide faced an elevated risk of adverse events (Table 2). These patients exhibited a higher prevalence of B‐lines on imaging and had elevated ACEF scores, both of which suggest a poor prognosis. Notably, these patients primarily relied on the Killip classification upon admission to assess pulmonary edema. However, this approach demonstrated limited sensitivity and often delayed the initiation of diuretic therapy, leading to adverse events during hospitalization. Interestingly, research by Claudia Marini [31] and Diego Araiza‐Garaygordobil [32] indicated that LUS‐guided diuretics reduced the risk of emergency visits related to worsening HF. However, no statistically differences were observed in hospitalization rates or all‐cause death among patients with HF. Our multivariate COX regression analysis yielded consistent results, showing that the use of diuretics and the occurrence of adverse events during the follow‐up period were not statistically significant. It is worth noting that pulmonary edema resulting from AMI is typically transient. To further evaluate the potential benefits, conducting large‐scale clinical studies to assess whether LUS‐guided diuretic use improves outcomes in AMI patients would be a valuable endeavor.

The ACEF score has been validated as a serviceable tool for predicting the prognosis of AMI patients after PCI [18]. In line with former studies by Side Gao et al. [33] and Barbara E Stahli et al., [19] our investigation demonstrated that the ACEF score is also effective for risk stratification in patients with AMI. However, our study stands out as the first to combine LUS with the ACEF score to evaluate the short‐term and medium‐term prognostic value in AMI patients. Our findings confirmed that both the ACEF score and the number of B‐lines detected by LUS were markedly associated with adverse cardiovascular events during hospitalization and the 1‐year follow‐up period. Importantly, when the ACEF score were combined with B‐lines, they provided enhanced predictive capability for AMI prognosis and improved risk stratification. Furthermore, LUS exhibited early identification of pulmonary edema in AMI patients with remarkable sensitivity and specificity [34, 35], making it a valuable tool for identifying high‐risk individuals.

The mortality and rehospitalization rates of patients with HF were as high as 15% and 30% within 2–3 months after discharge, a period often referred to as the vulnerable phase of HF [36, 37, 38]. Unfortunately, there is currently no effective method to reliably identify high‐risk patients during this critical time. In our study, we investigated whether combining B‐line assessment with the ACEF score could enhance risk stratification during the first 3 months of follow‐up. Our findings revealed that patients in the low‐risk group (based on B‐line and ACEF score) experienced no adverse events, while the high‐risk and medium‐risk groups faced markedly increased risks. However, further research is needed to determine whether this combined approach can effectively discover high‐risk patients during the vulnerable phase of HF.

Our study findings indicate that the presence of B‐lines upon hospital admission is a predictive marker for the severity of pulmonary edema. Furthermore, it correlates with an increased risk of adverse events during follow‐up and hospitalization. Timely identification of pulmonary congestion using LUS, which surpasses lung auscultation in sensitivity, holds promise for improving the short‐ to medium‐term prognosis in patients with AMI. Furthermore, the ACEF score—incorporating patient age, EF, and creatinine levels—provides valuable insights into the long‐term prognosis of patients with AMI. Our integrated research combining radiological imaging and laboratory tests demonstrates that both LUS and the ACEF score complement each other as prognostic indicators in patients with AMI. Utilizing both LUS and the ACEF score provides valuable clinical insights, aiding in predicting the near‐ to medium‐term prognosis after PCI and identifying high‐risk patients. Nevertheless, additional research is necessary to establish optimal management strategies for high‐risk patients.

### 4.1 Limitation

Our study is conducted at a single center and involves a limited sample size from a Chinese population. As a result, the generalizability of our findings is restricted. Additionally, the follow‐up period was brief, primarily conducted via telephone, and some patients lacked outpatient follow‐up data. Further extensive independent studies are essential to establish a robust association between combining the ACEF score with the number of B‐lines and risk stratification in patients with AMI. Furthermore, it's worth noting that some AMI patients faced emergency conditions and didn't have sufficient time for LUS assessment before PCI. All patients eventually underwent LUS within 48 h of the perioperative duration, which might have marginally influenced our data and results. Lastly, due to the patient's supine position upon admission, we were unable to measure height and weight, precluding the calculation of body mass index (BMI). This limitation could potentially impact the adjustment of particular indicators.

## 5 Conclusion

In summary, LUS and the ACEF score were associated with adverse cardiovascular events during follow‐up and hospitalization. Both LUS and the ACEF score demonstrated good predictive value for the short‐term and medium‐term prognosis of AMI patients. Furthermore, their combination provided incremental value for prognosis evaluation. Therefore, utilizing LUS in conjunction with the ACEF score can effectively serve as an assessment tool for patients with AMI.

## Author Contributions

ZL, MF, SY, and ZL conducted data collection, analysis, and material preparation. ZL authored the initial draft of the manuscript, and all authors provided feedback on earlier versions. HD and YZ share equal responsibility for ensuring the accuracy and integrity of all aspects of the work, addressing related questions, and resolving them appropriately. All authors contributed to the research design and conception, and unanimously approved the final manuscript.

## Ethics Statement

The research involving human participants underwent was reviewed and approved by the Ethics Committee of Guangdong Provincial People's Hospital. All participants/patients provided written informed consent.

## Conflicts of Interest

The authors declare no conflicts of interest.

## Supporting information

Supporting information.

## Data Availability

The authors will provide the unanalyzed data, which will make it possible to draw conclusions from this article without any undue reservations.
