# Supplementary material for: Predictive Value of Lung Ultrasound Combined With ACEF Score for the Prognosis of Acute Myocardial Infarction
Source: Clin Cardiol. 2025 Feb 3;48(2):e70077. doi: 10.1002/clc.70077 (PMC11790606; doi:10.1002/clc.70077)
Supplement: Supplementary file 1 — Supporting information. [file CLC-48-e70077-s001.docx]

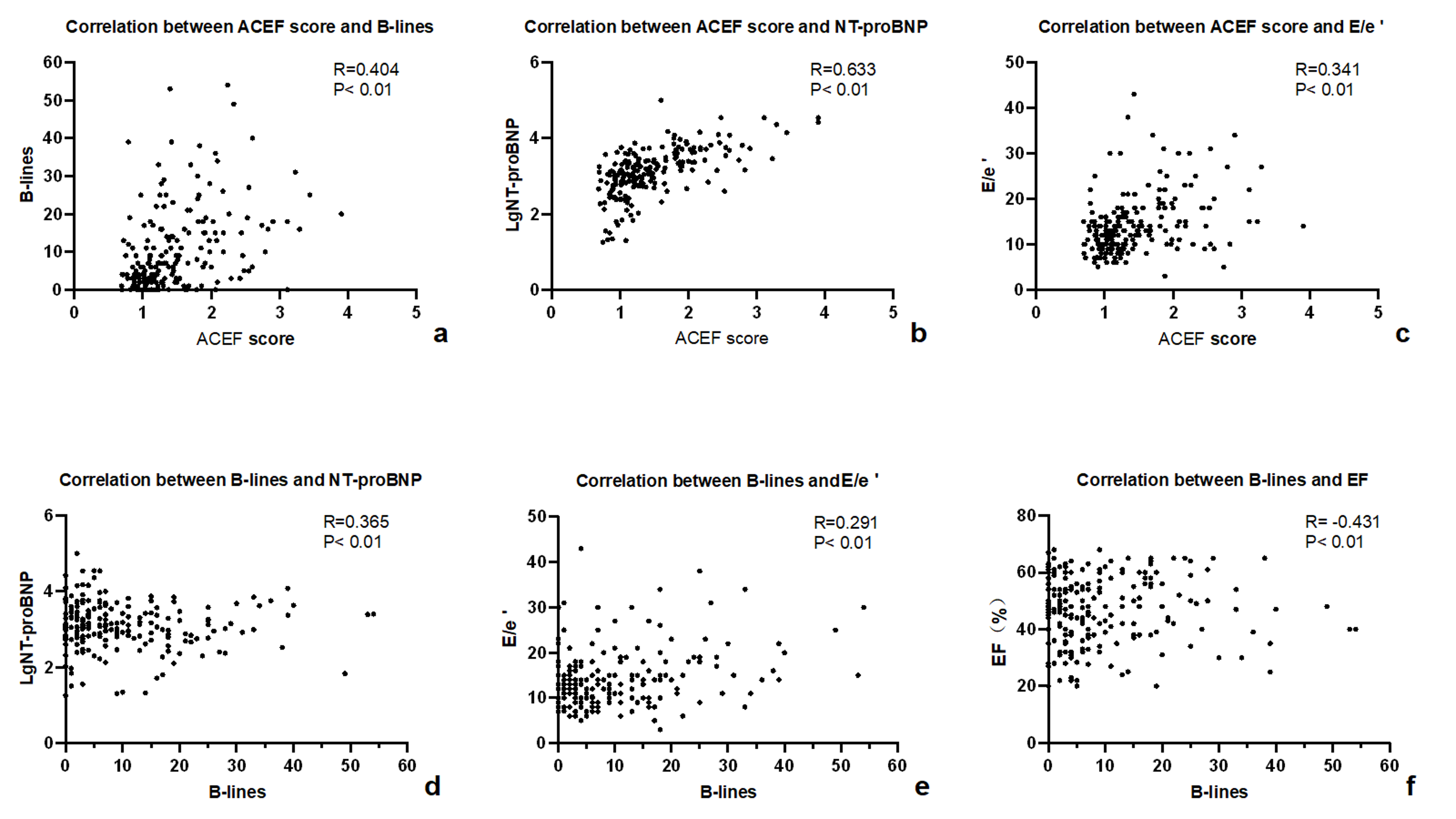


Supplemental Fig.1 Scatter plot of correlation: a. Correlation between ACEF score and B-lines; b. Correlation between ACEF score and NT-proBNP; c. Correlation between ACEF score and E/e'; d. Correlation between B-lines and NT-proBNP; e. Correlation between B-lines and E/e'; f. Correlation between B-lines and EF.

Supplemental Table 1. Univariate Analysis during Hospitalization and Follow-up

|  | Univariate analysis during hospitalization | | Univariate analysis during follow-up | |
| --- | --- | --- | --- | --- |
|  | OR（95%CI） | P value | HR（95%CI） | P value |
| Age（Years） | 1.05(1.02-1.08) | 0.01 | 1.03(1.01-1.06) | 0.02 |
| Smoking (previous or current) | 0.65(0.40-1.18) | 0.16 | 0.67(0.38-1.17) | 0.16 |
| Previous PCI | 1.00(0.33-3.01) | 1.00 | 0.47(0.12-1.95) | 0.30 |
| Diabetes | 2.08(1.08-3.98) | 0.03 | 1.35(0.74-2.47) | 0.33 |
| Cardiac shock | 17.72(3.86-81.23) | 0.01 | 2.21(0.88-5.58) | 0.09 |
| Systolic blood pressure (mmHg) | 0.96（0.95-0.98） | 0.01 | 0.99(0.98-1.00) | 0.20 |
| Furosemide | 10.79(5.38-21.63） | 0.01 | 2.85(1.61-5.02) | 0.01 |
| Number of vessels | 1.49（1.10-2.01） | 0.01 | 1.45(1.09-1.93) | 0.01 |
| Number of B lines | 1.13（1.09-1.18） | 0.01 | 1.07(1.05-1.10) | 0.01 |
| ACEF score | 9.76（4.76-20.00） | 0.01 | 3.03(2.09-4.38) | 0.01 |
| logTNT | 1.36（0.88-2.10） | 0.17 | 1.48(0.92-2.37) | 0.10 |
| logNT-BNP | 9.51(4.42-20.48) | 0.01 | 2.21(1.41-3.48) | 0.01 |
| Creatinine (mg/dl) | 1.01(1.00-1.02) | 0.02 | 1.00(1.00-1.01) | 0.02 |
| Killip classification | 2.69(1.91-3.79) | 0.01 | 1.50(1.17-1.93) | 0.01 |
| E/e’ ratio | 1.09(1.03-1.14) | 0.01 | 1.02(0.97-1.06) | 0.46 |
| LA (mm) | 1.05(0.99-1.11) | 0.11 | 1.07(1.02-1.13) | 0.01 |
| EF (%) | 0.90(0.87-0.93) | 0.01 | 0.95(0.92-0.97) | 0.01 |

PCI, percutaneous coronary Intervention; Log NT-proBNP, log-transformed N-terminal pro-brain natriuretic peptide; Log TNT, log-transformed troponin T; LA, left atrium; ACEF score: Age, creatinine, and ejection fraction score; EF, ejection fraction. OR, odds ratio; HR, hazard ratio.
